# The latest evidence and guidance in lifestyle and surgical interventions to achieve weight loss in people with overweight or obesity

**DOI:** 10.1111/dom.16296

**Published:** 2025-03-03

**Authors:** Iskandar Idris, Oluwaseun Anyiam

**Affiliations:** ^1^ Centre of Metabolism Ageing & Physiology, School of Medicine University of Nottingham Nottingham UK; ^2^ East Midlands Bariatric Metabolic Institute (EMBMI) University Hospitals Derby & Burton Foundation Trust Derby UK

**Keywords:** Bariatric, evidence, guidance, lifestyle, metabolic, obesity, surgery, weight loss

## Abstract

**Background:**

The prevalence of obesity and related co‐morbidities has reached epidemic proportions. Effective evidence‐based treatment approaches are therefore important. Lifestyle intervention remains the mainstay of the treatment strategy to manage obesity. Increased evidence has also emerged regarding the efficacy of metabolic bariatric surgery (MBS) to induce significant and sustained weight loss while also reducing the progression of obesity‐related co‐morbidities for people living with obesity.

**Aims & Methods:**

This article aims to bring together current evidence, guidance and best practice for the prevention and management of people living with overweight or obesity by means of lifestyle and behavioural intervention, as well as by MBS.

**Result:**

Lifestyle intervention encompasses dietary strategies, physical activity and behavioural intervention. Discussion on MBS will focus on current indications, comparison between different MBS procedures, novel endoscopic techniques, potential complications and pre‐operative management.

**Plain Language Summary:**

The number of people living with excess weight and complications associated with being overweight is alarmingly quite high. Effective treatment approaches that are supported by clinical studies are therefore important. Lifestyle changes remain very important to manage excess weight. Increased evidence has also shown the benefits of weight loss surgery to produce significant weight loss which could be sustained, while also reducing the risk of developing medical conditions associated with excess weight. This article aims to bring together current evidence, guidance and best practice for the prevention and management of people living with excess weight by means of lifestyle and behavioural changes, as well as by weight loss surgery. Lifestyle intervention encompasses dietary strategies, physical activity and behavioural intervention. Discussion on weight loss surgery will focus on current criteria for suitability, comparison between different weight loss surgery procedures, new techniques, possible complications and appropriate management prior to weight loss surgery.

## 1 INTRODUCTION

Obesity is a chronic multifactorial disease influenced and regulated by a complex interplay of hormones and a cascade of adaptive metabolic and physiological mechanisms that are central to the disease process.^1^ The prevalence of obesity, defined as a Body Mass Index (BMI) of greater than or equal to 30 kg/m^2^ has nearly tripled in numbers since 1975.^2^ Recent data from the World Health Organization (WHO) has estimated that in 2022, 1 in 8 people worldwide were living with obesity, while 43% of adults were classified as overweight.^3^ Alarmingly, over 390 million children and adolescents aged 5–19 years were overweight, including 160 million who were living with obesity.^3^ An ‘obesogenic’ environment with increasing per‐capita food supplies, sedentary lifestyles, genetics and the gut microbiome are among many other factors contributing to the rising epidemic.^4^

Weight loss, even modest, has been shown to significantly lower the risk of cardio‐metabolic, respiratory, gastrointestinal and musculoskeletal disorders, to name a few, and also improve quality of life.^5^ Various strategies have therefore been developed not only to curb the rising tide in the prevalence of obesity, but also to manage individuals living with complex severe obesity. Lifestyle (dietary, behavioural and physical activity) interventions remain the cornerstone of prevention and treatment strategies, while weight loss surgery (metabolic and bariatric surgery, MBS) is a treatment of choice for suitable patients. Long‐term weight loss maintenance, however, remains an ongoing challenge to individuals living with overweight/obesity.

This article will focus on current evidence, guidance and best practice for the prevention and management of people living with overweight or obesity by means of lifestyle and behavioural intervention, as well as by MBS. Data search was conducted from MEDLINE & EMBASE, PubMed and Google Scholar, Clinical trials.gov. A thematic assessment of relevant publications, guidelines and position statements was undertaken and selected to cover the remit of the review, that is, to cover ‘The Latest Evidence, Guidance and Best Practice in Lifestyle & Surgical Interventions To Achieve Weight Loss in People With Overweight or Obesity’.

## 2 LIFESTYLE INTERVENTION

Lifestyle interventions encompass dietary, physical activity and behavioural modifications to induce weight loss. The complex interplay between behavioural, biological, cultural, economic, environmental and psycho‐social factors needs to be considered during such interventions, as these factors play a crucial role not only in the effectiveness of inducing weight loss but also in maintaining weight loss. Central to lifestyle intervention are strategies to reduce energy intake, coupled with high levels of physical activity (e.g., prescribed aerobic exercise and resistance training), promoting active leisure‐time pursuits and reduced sedentary time, all of which aim to induce and maintain meaningful weight loss – often defined as a reduction of >5% of initial weight, a target weight loss known to be associated with cardio‐metabolic benefits.^6^

In 2018, the United States Preventive Services Task Force (USPSTF) reaffirmed its recommendation that primary care health professionals screen all adults for obesity and offer those affected ‘intensive, multicomponent behavioural interventions’^7^ This recommendation reflect the strength of evidence that supports the efficacy of behavioural intervention, described in this review for the management of obesity. However this is likely to be challenging to deliver, due to the vast number of people living with obesity as well as the limited expertise to deliver this.

The current most comprehensive lifestyle guidelines are derived from two sources — the American College of Cardiology (ACC)/American Heart Association (AHA)^8^ and the American Association of Clinical Endocrinologists (AACE)/American College of Endocrinology (ACE).^9^ These guidelines recommend that individuals participate in a comprehensive intervention for at least 6 months, delivered by trained health care professionals including registered dietitians, psychologists or health counsellors, as well as, where appropriate, lay persons who are appropriately trained to deliver lifestyle and behavioural interventions that are designed to modify dietary intake and physical activity.^8^, ^9^ These interventions are typically multi‐disciplinary, supporting patients to pursue behavioural interventions that support adherence to physical activity and meal plan prescriptions. This can include activities such as goal setting, self‐monitoring of food intake and physical activity, 1:1 sessions with clinicians (e.g., to deliver cognitive behavioural therapy, dietary education) and group meetings (e.g., gatherings with peers, use of social support structures).^9^ A remote approach to support weight loss has also been shown to be effective. The use of a Wireless feedback system (WFS) including a Wi‐Fi activity tracker and scale transmitting data to a smartphone app to provide daily feedback on progress in lifestyle change and weight loss, for example, has been shown to be as effective to induce significant weight loss. In this study, participants' self‐monitoring data was viewed on a dashboard; and step‐up interventions which included supportive messaging via mobile device screen notifications (app‐based screen alerts) without or with coaching or powdered meal replacement was provided where appropriate.^10^ In another study, participants underwent an automated online weight loss (WL) program (4 months) and WL maintenance program (8 months), consisting of video lessons, self‐monitoring and personalized feedback. Individuals with suboptimal responses who received brief or extended telephone coaching experienced greater weight loss than control, with weight loss reported to be 6.2%–7% of baseline.^11^

Irrespective of the approach, frequent contact is crucial for effective weight loss. As such, in the first 6 months of a lifestyle intervention, individuals should receive at least 14 individual or group treatment sessions. Such high‐intensity programs produce approximately 5% to 10% body weight loss, on average, over 6 months, with no additional benefits observed by increasing intensity further (e.g., 24 rather than 16 sessions in 6 months).^12^ A recent meta‐analysis indicated that online, group‐based interventions would also produce a statistically significant impact on weight loss in people with severe obesity, but barriers such as internet accessibility, digital literacy and unfamiliarity with group members need to be mitigated.^13^ Cost effectiveness may favour the use of group rather than individual treatment, with a recent systematic review reporting greater efficacy with group treatment^14^ but the approach needs to be individualized according to patients' preferences. Where patients fail to achieve >2.5% weight loss within 1 month of starting treatment, behavioural interventions need to be escalated.^10^ Maintenance of weight loss is challenging, with many individuals affected by weight regain. The reasons for this are multifactorial, which include physiological and psychological factors, adherence to dietary and lifestyle interventions, socio‐economic factors, and the impact of comorbidities as well as the use of concurrent therapies that may promote weight gain. Clinical assessments should therefore include identification of obesity‐related comorbidities using the AACE/ACE guideline^15^ or the Edmonton Obesity staging system^9^ (Table 1) and readiness to make change. The presence of comorbidities should individualize treatment targets, that is, a minimum of 5% weight loss for obesity stages 1 & 2; and at least 10% weight loss for individuals with higher stages of obesity.

**TABLE 1:** Edmonton obesity staging system.

Stage 1	Stage 2
**Subclinical** risk factors with **mild** symptoms such as borderline hypertension, pre‐diabetes or mild osteoarthritis	Established comorbidities requiring medical treatment with **moderate** symptoms such as depression, HTH, T2DM, GERD, OSA, Fatty liver disease

Stage 3	Stage 4
Significant obesity‐related comorbidities with **significant** limitations, end organ damage or impairment such as myocardial infarction, suicidal ideation, reduced mobility, stroke and diabetic complications	**Severe** (i.e., end‐stage) disabling symptoms and limitations such as wheelchair or bed‐bound, pulmonary hypertension from OHS, end‐stage renal disease from diabetic nephropathy, and decompensated cirrhosis from NASH

### 2.1 Dietary component

To promote weight loss, current guidelines recommend dietary restriction to achieve a calorie deficit of approximately 500–700 kcal/ day, with the aim to induce a mean loss of 0.5–0.75 kg (1.0–1.5 lb) per week.^8^, ^9^ This proposed calorie deficit and rate of weight loss is a guide, designed to avoid sudden changes in calorie intake which can lead to adverse health effects and may even result in long‐term weight gain. In the Look AHEAD trial, a randomized controlled trial comparing an Intensive Lifestyle Intervention to a Diabetes Support and Education in overweight and obese type 2 diabetes patients to track the development of cardiovascular disease over time, 1200–1500 kcal/d was prescribed for individuals who weigh <113 kg and 1500–1800 kcal/d for those >113 kg.^16^ Reducing portion size is a useful strategy but is less effective if the food eaten is still energy dense. The overall aim is to increase low‐energy dense foods such as salad and vegetables, whilst concurrently decreasing the amount of high‐energy dense macronutrients such as carbohydrates and fats. By reducing overall energy intake, alongside ensuring adequate intake of complex carbohydrates (e.g., starches and dietary fibres) and consuming more salad and vegetables, satiety can be improved despite ingesting less overall energy.

Estimating the proportion of macronutrients contributing to overall intake is a useful starting point. The US dietary guidelines recommend that approximately 15%–35% of daily energy are derived from protein, 20%–35% from fat (with no more than 10% from saturated fat) and 45%–65%r from carbohydrates.^17^ The focus is to customize and enjoy nutrient‐dense food and beverage choices to reflect personal preferences, cultural traditions and budgetary considerations but to stay within energy limits.^17^ While there is little evidence that macronutrient composition per se affects weight loss independent of energy restriction, a recent study showed that increasing protein at the expense of fat or carbohydrates, and reducing starch by increasing other macronutrients, might be associated with increased weight and waist gain.^18^

European Society of Cardiology (ESC) guidelines for cardiovascular prevention in clinical practice^19^ is another important body that provides updated evidence‐based guidance for the management of weight loss for people living with obesity. The key dietary principles recommended by ESC include adopting a plant‐based diet rich in whole grains, vegetables, fruits, nuts and fibre‐rich foods, while replacing saturated fats with unsaturated fats, and reducing salt and sugar (especially sugary beverages) and alcohol consumption.^19^

Beyond energy restriction, the ESC endorses a variety of dietary approaches including low or very‐low carbohydrate diets (50–130 g and 20–49 g carbohydrate per day, respectively), low fat diet (<30% of energy from fat), high protein diets, Mediterranean‐style diets, low‐glycaemic‐load diets and time‐restricted eating.^19^ All of these interventions can induce weight loss if they facilitate the achievement of desired energy deficits; however, although they result in similar short‐term weight loss, only the Mediterranean diet is the only dietary pattern approach with an adequately sized and powered RCT supporting its long‐term benefits. This has since been superseded by the Very Low energy Diet approach to be discussed later. Low‐ or very‐low carbohydrate diets and the ketogenic diet have been widely studied as an approach to induce weight loss in people with or without diabetes. A Cochrane review, which included 61 parallel‐arm RCTs involving 6925 overweight or obese participants with or without diabetes, however, reported little to no difference in weight reduction and changes in cardiovascular risk factors up to 2 years' follow‐up between low‐carbohydrate or balanced‐carbohydrate weight‐reducing diets.^20^ High protein diets can help maintain lean muscle mass and promote satiety.

With regards to time‐restricted eating, an RCT randomly assigned 139 patients with obesity to time‐restricted eating (eating only between 8:00 AM and 4:00 PM) with energy restriction or daily energy restriction alone. No difference was observed between the groups with regards to reduction in body weight, body fat or metabolic risk factors.^21^ A recent meta‐analysis has also reported that intermittent fasting (e.g., time restricted eating, 5:2 diet or alternate day fasting) is comparable to a traditional energy restriction diet with regards to weight loss. Alternate day fasting, however, showed the highest effectiveness for weight loss, followed by traditional energy restriction and time‐restricted eating.^22^ Further well‐powered RCTs with longer durations of intervention are required to draw solid conclusions, but overall evidence suggests the need to use dietary intervention strategies that are able to induce and maintain energy restriction.

In contrast to the varying levels of certainty in the efficacy of different dietary strategies discussed above, the strength of evidence for the efficacy and safety of very‐low energy diets (VLED) for the treatment of obesity and type 2 diabetes is now widely established. By limiting daily energy intake to less than 800 kilocalories (kcal), VLED interventions – usually by means of Total Dietary Replacement – result in approximately 13–18% weight loss. Furthermore, in the context of type 2 diabetes, VLED can induce diabetes remission. The DIRECT study was an open‐label, cluster‐randomized trial at 49 primary care practices aimed at assessing whether intensive weight management within primary care would achieve remission of type 2 diabetes.^23^ The intervention comprised the withdrawal of antidiabetic drugs and a total diet replacement (825–853 kcal/day) formula diet for 3–5 months. This was provided by soups and shakes produced by the Cambridge Weight plan. The 12‐week period is followed by a stepped food reintroduction (for 2–8 weeks) followed by a weight loss maintenance phase where participants were advised to follow a food‐based diet and were provided with an individually tailored energy prescription to support weight stabilization and prevent weight regain. At 12 months, mean body weight fell by approximately 10 kg in the intervention group and 1.0 kg in the control group, whilst diabetes remission was achieved in 46% and 4%, respectively. The remission rate, however, was reduced to 36% at 2 years and at 5 years, 13% remained in remission.^24^ At 5 years, average weight loss in the intervention group was 5.6 kg and 4.6 kg in the control group. Following publication of the original DIRECT study, this dietary intervention programme has been replicated in real‐world practice. In 2019, the English National Health Service (NHS) established a total dietary replacement‐based interventional programme within a real‐world environment, known as the NHS Type 2 Diabetes Path To Remission Programme.^25^ This involves a 12‐month behavioural intervention to support weight loss involving an initial 3‐month period of total dietary replacement. The mean weight loss for the 1710 participants who started the programme was 8.3% (~9.4 kg) and the mean weight loss of the 945 participants who completed the programme was 9.3% (~10.3 kg). Among the latter group, 32% (*N* = 145) achieved diabetes remission at 1 year. The rate of remission in a real‐world setting is lower than that observed in the randomized controlled trial.^25^ Further research into strategies to maintain weight loss and diabetes remission over the longer term is required.

### 2.2 Physical activity

Where possible, physical activity needs to be implemented alongside dietary modifications to support weight loss.^8^, ^9^ The ACC/AHA guideline recommends at least 150 min of aerobic physical activity (e.g., brisk walking) per week (equivalent to 30 min per day for 5 days of the week) for initial weight loss, increasing to approximately 200 to 300 min per week to prevent weight regain.^8^, ^9^, ^26^ Each 30 min per week of aerobic exercise has been shown to be associated with reduced body weight by 0.52 kg (95% CI, −0.61 to −0.44 kg; *n* = 109 trials); waist circumference by 0.56 cm (95% CI, −0.67 to −0.45 cm; *n* = 62 trials); body fat percentage by 0.37% (95% CI, −0.43% to −0.31%; *n* = 65 trials), as well as the areas of visceral and subcutaneous adipose tissues loss.^26^ Evidence supports combining aerobic and resistance exercise, along with weight loss to preserve the loss of lean mass, especially in older adults with obesity.^27^ Individualized exercise regimes and goal settings need to be considered due to challenges such as sarcopenic obesity and/or frailty in the older age groups.

### 2.3 Behavioural component

An interesting approach to lifestyle intervention is one that advocates interventions that address the underlying pathophysiology and behavioural features of obesity, personalized to the most predominant underlying pathogenic factor of the individual. Three phenotypic domains have been suggested: homeostatic eating, hedonic eating behaviour and abnormal energy expenditure.^28^ These domains can be further categorized into four actionable phenotypes: abnormal satiation (measured by calories ingested to experiencing postprandial fullness), abnormal postprandial satiety (duration of fullness), emotional eating behaviour and abnormal resting energy expenditure. Previous studies have shown that these phenotypes could explain 85% of the variance in obesity.^28^ A proof‐of‐concept study evaluated the outcomes of a phenotype‐tailored lifestyle intervention on weight loss, cardio‐metabolic risk factors and physiologic parameters in adults with obesity. Phenotype‐tailored diet was derived from several nutritional studies that showed targeted‐specific physiological or metabolic benefits after/during a specific intervention and is summarized in Table 2. At the end of the 12 weeks intervention, the phenotypic lifestyle intervention resulted in a significant weight loss of −7.4 kg compared with control of −4.3 kg, with no adverse events reported.^29^ The study forms a basis for the need for an RCT to confirm causality. Importantly, it provided good evidence of an objective, systematic approach to individualized behavioural intervention strategies to induce weight loss. This is important since the aetiology of obesity is heterogenous, with social, cultural, psychological and physiological factors playing an important role in the effectiveness of weight loss intervention strategies for individual patients.

**TABLE 2:** Phenotypic based dietary intervention (#Reference 28).

Phenotype	Characteristic	Aim	Example
***Abnormal satiation***	Abnormal fullness	The intervention aimed to keep the brain hunger centre ‘switched off’ for longer periods of time, by reducing the allowed period of caloric intake during a day; the intervention was also tailored to produce maximal gastric distension to induce the sensation of fullness	Using a volumetric diet, and if desired, a healthy second serving of fruits or vegetables, helping participants to reach satiation.
***Abnormal postprandial satiety***	Accelerated gastric emptying and increased post prandial hunger	Increase endogenous GLP‐1 production to delay gastric emptying	To deliver protein preloads to increase the early release of gastrointestinal hormones, delaying gastric emptying.
***Abnormal emotional eating***	Negative mood, high anxiety and reward‐seeking behaviours in relation to negative and positive emotions	Anxiety is highly correlated with negative perceptions to food and emotional eating	Behavioural intervention structured to improve emotional regulation, self‐efficacy, goal‐setting, self‐monitoring and stimulus control through the use of a targeted mindfulness‐based motivational approach
***Abnormal resting energy expenditure (REE)***	Reduced REE, and muscle mass	Low REE was suggested to play a role in the development of obesity, contributing toward positive energy balance and subsequent weight gain. Body composition is the most important driver of REE, particularly in metabolically active tissues such as lean mass	Intervention based on a structured exercise plan to increase muscle mass, to increase overall energy expenditure ratio; implement a high‐intensity resistance training to enhance muscle strength and size to boost total energy expenditure, and with a protein supplement post‐exercise for muscle mass.

Mindfulness‐based interventions (MBIs) targeting eating behaviours have also gained popularity in recent years. Such interventions used a variety of approaches to implement mindfulness training, including combined mindfulness and cognitive behavioural therapies, mindfulness‐based stress reduction, acceptance‐based therapies, mindful eating programmes and combinations of mindfulness exercises. A previous literature review was conducted to determine the effectiveness of MBIs for treating obesity‐related eating behaviours, such as binge eating, emotional eating, external eating and physical activity participation in adults with overweight and obesity.^30^ It examined a variety of approaches to implement mindfulness training, such as combined mindfulness and cognitive behavioural therapies, mindfulness‐based stress reduction, acceptance‐based therapies, mindful eating programmes and combinations of mindfulness exercises.^30^ The review suggests that mindfulness training has short‐term benefits on health‐related behaviours. The only significant predictor of weight loss was follow‐up distance from post‐intervention, that is, the longer follow‐up distances were associated with greater weight loss. A subsequent RCT assesses the effect of Mindful eating associated with moderate energy restriction on weight loss in women with obesity. The study reported a greater reduction in uncontrolled eating with mindful eating and emotional eating with mindful eating intervention.^31^ Application of mindfulness‐based eating behaviour strategies, taught at group sessions within a tier 3 obesity service in a United Kingdom centre, has also reported significant improvement in eating behaviour and facilitated subsequent weight loss over 6 months.^32^ Future studies should explore the effectiveness of mindfulness training on long‐term post‐intervention weight loss in adults with overweight and obesity.

## 3 METABOLIC BARIATRIC SURGERY (MBS)

Metabolic Bariatric surgery (MBS) remains the gold standard intervention for long‐term weight loss and management of obesity. It results in a mean peak weight loss between 30% and 35% and long‐term weight loss of approximately 25%.^33^ Between 20 and 35% of patients, however, experience suboptimal weight loss and previous analyses indicate that weight loss outcomes are dependent on the choice of surgery, age, initial BMI, ethnic origin, presence of eating disorders, metabolic factors and compliance with post‐surgical dietary guidance.^34^

The latest International Federation for Surgery for Obesity and Metabolic Disorders (IFSO) report estimate suggests at least half a million bariatric procedures were performed worldwide in 2023, with the most being performed in the USA and Brazil.^35^ Data from the American Society for Metabolic and Bariatric Surgery (ASMBS) show an increase in bariatric procedures performed, with 100,000 more procedures performed in 2022 than 10 years prior.^36^

### 3.1 Types of MBS procedure

**FIGURE 1:**
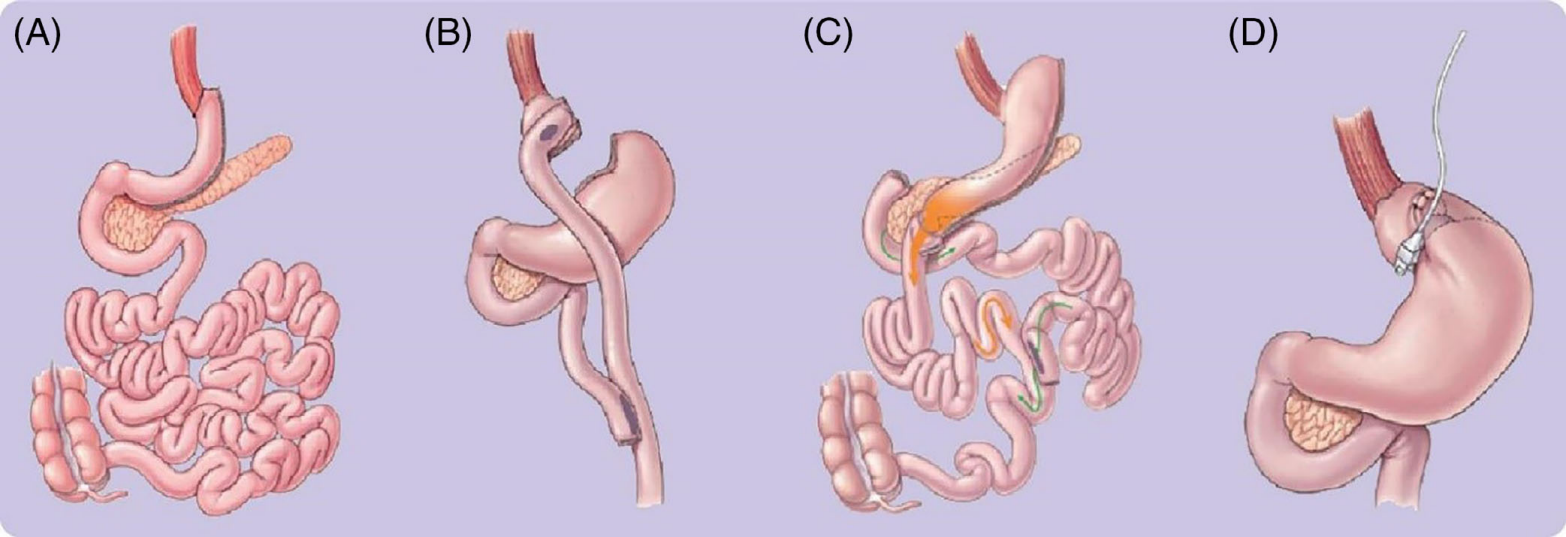
Metabolic Bariatric surgery procedures: A Sleeve gastrectomy B Roux‐en‐Y gastric bypass C biliopancreatic diversion; D Adjustable gastric banding. Adapted from American Society for Metabolic and Bariatric Surgery (https://asmbs.org/patients/bariatric‐surgery‐procedures).

Multiple bariatric procedures exist, and the most widely described types are (see Figure 1):

- Sleeve gastrectomy
- Roux‐en‐y gastric bypass (RYGB)
- Adjustable gastric band
- One‐anastomosis gastric bypass (OAGB)
- Biliopancreatic diversion

By far the most common procedure performed worldwide is the sleeve gastrectomy^35^, ^36^ which now comprises approximately 60% of all bariatric surgical procedures. RYGB is the next most common type, and 9 out of every 10 bariatric procedures will be one of these two types.^35^ The remainder are divided equally between OAGB (approximately 4.3%) and other less common procedures.^35^

Whilst the adjustable gastric band was a popular choice in the early part of the previous decade, the number of these performed has dramatically reduced in recent times.^36^ Open procedures predominated at the beginning of the last decade; however, laparoscopic approaches are currently the more preferred modality, largely owing to a shorter length of hospital stay and a reduced risk of postoperative complications.^37^

The sleeve gastrectomy involves the removal of the majority of the stomach, leaving a narrow vertical ‘sleeve’ of stomach with a reduction in volume of up to 75%.^38^ The strength of this procedure lies in the fact that, unlike most other types of bariatric surgery, it only involves the stomach, enabling a more rapid operation with lower potential for intraoperative complications.^37^ In contrast, the RYGB is a multistep procedure with the initial division of a smaller upper section of the stomach, connection of this smaller pouch directly to the small intestine (bypassing the duodenum and proximal ileum), closure of the larger body of the stomach which remains in situ, and anastomosis of the ‘gastric limb’ to a more distal part of the small intestine.^36^ Thus, two anastomoses are formed, resulting in the formation of a Y‐shaped system.

The OAGB is a simplified form of bypass procedure, in which a longer pouch of stomach is created and anastomosed directly to a distal part of the small intestine, while the connection between the stomach, duodenum, and ileum remain anatomically intact.^38^ The adjustable band is the simplest procedure in which a silicone ring is inserted around the proximal portion of the stomach, reducing its capacity to receive food, promoting early satiety and inducing weight loss.^39^ Within this ring is a balloon that is connected to an external port, enabling subsequent alteration to modulate food intake without the need for further invasive procedures. The reduced efficacy compared with other procedures, along with the need for frequent adjustments and relatively high chance of conversion to other procedures, has led to this procedure becoming less commonly performed.^37^

### 3.2 Indications for MBS

Guidance published jointly by ASMBS and IFSO recommends MBS in any individual with a BMI ≥35 kg/m^2^, regardless of the presence, absence or severity of weight‐related comorbidities.^40^ Additionally, MBS should be considered for individuals with BMI 30–34.9 kg/m^2^ and the existence of metabolic disease. Adjustments should be made in the Asian population, such that individuals of this ethnicity should be considered for bariatric surgery if BMI ≥27.5 kg/m^2^.^40^ Notably, this differs slightly from European and United Kingdom guidance, which recommends MBS for individuals with BMI ≥40 kg/m^2^, or BMI 35–39.9 kg/m^2^ accompanied by a significant health condition that could be improved with weight loss.^41^, ^42^ There is an additional consideration for individuals with BMI 30–34.9 kg/m^2^ who have coexisting type 2 diabetes.^41^, ^42^ UK guidance also stipulates that assessment for suitability of bariatric surgery must be performed in a specialist weight management service and reduction of BMI limits by 2.5 kg/m^2^ should be applied for individuals from ethnic minority backgrounds.^41^

### 3.3 Efficacy of MBS versus non‐surgical weight management

Numerous studies have successfully demonstrated superior efficacy with MBS compared with intensive non‐surgical interventions, in the short^43^, ^44^ and long term.^45^, ^46^, ^47^ A systematic review and meta‐analysis of randomized controlled trials reported that all studies comparing bariatric surgery to intensive medical interventions observed greater weight loss, regardless of the procedure utilized.^48^ Surgical interventions resulted in a mean 22.05 kg superior weight loss across included studies. There were also associated improvements in outcomes related to total cholesterol, triglycerides, systolic blood pressure, HbA1c, HOMA‐IR and cardiovascular risk.^48^

Despite the strength of evidence favouring MBS compared with non‐surgical methods, the number of RCTs comparing non‐surgical and surgical treatment is small, and most of them only follow up in the short term. In addition, there is variability in study designs, and many studies do not adequately describe the strategy used in non‐surgical treatment. This lack of data and standardization in this type of treatment can lead to bias and possibly the formation of extremely heterogeneous groups for analysis. In addition, the majority of studies have included diabetes as an inclusion criterion, and hence findings may not be generalized to patients with obesity without diabetes. Furthermore, the optimal treatment option is dependent on individual patient characteristics, and as such, the impact on quality of life is likely to be subjective and difficult to assess. Finally, it is important to undertake a robust RCT comparing MBS with more novel weight loss treatments such as tirzepatide to assess not only weight and metabolic outcomes but also health economics and quality of life outcomes.

### 3.4 Comparisons between bariatric procedures

The choice of procedure generates considerable debate, and several trials have been conducted to perform efficacy and safety comparisons between the types of surgery. The most widely studied is the comparison between sleeve gastrectomy and RYGB, with inconsistent results reported. Two noteworthy RCTs performed in 2018 highlight this incongruence. SLEEVEPASS randomized 240 patients to one of the two procedures and demonstrated significantly greater 5‐year excess weight loss with RYGB, along with greater resolution of co‐existing hypertension.^49^ However, the SM‐BOSS study failed to replicate this difference in 5‐year weight outcomes.^50^ There was also no significant difference in hypertension resolution observed in SM‐BOSS; however, significantly greater resolution of gastro‐oesophageal reflux disease (GORD) and some dyslipidaemia markers did occur in the RYGB group. Furthermore, whilst SM‐BOSS did not appreciate any significant difference in early or late complication occurrence, RYGB was associated with a significant increase in early complications in SLEEVEPASS.^49^, ^50^

More recently, the SleeveBypass study reported a large RCT in which 628 patients were randomized between the two procedures to address the question.^51^ Similar to SLEEVEPASS, significantly greater weight loss was demonstrated with RYGB, associated with greater improvements in dyslipidaemia.^51^ However, a significantly greater incidence of minor complications occurred with RYGB, although there was no difference in major complications, and an increase in the development of GORD following sleeve gastrectomy.^51^ Another recent RCT of 1735 patients did not report weight outcomes, instead focusing on perioperative data and 90‐day mortality. This study demonstrated significantly shorter operative time with sleeve gastrectomy, with no significant difference in overall adverse events.^52^ The discrepancies between these studies may have occurred due to a variety of reasons, for example, differences in the population being studied (e.g., proportion of females, % diabetes, ethnic group, etc.), non‐standardized surgical procedures and operating techniques, and differences in the health care system in different countries, etc. These studies are summarized in Table 3.

**TABLE 3:** Summary of the 4 studies comparing Sleeve Gastrectomy with Roux‐en‐Y Gastric bypass.

Author	Country	Patient characteristics	Age	% female	BMI at baseline (SG v RYGB)	Follow up	Evaluated comorbidities	Weight outcome	Other outcomes
Salminen P et al.^49^	Finland	Age 18–60, BMI >40 or >35 with significant comorbidity 42% have diabetes	48	69.9	45.9 (48.5 v 48.4)	5 years	Type 2 diabetes, dyslipidaemia, hypertension	Mean % excess weight loss at 5 years was 49% after sleeve gastrectomy and 57% after RYGB	Complete or partial remission of type 2 diabetes 37% after sleeve gastrectomy and 45% after RYGB (*p* > 0.99). Resolution of dyslipidaemia in 47% after sleeve gastrectomy and 60% (RYGB) (*p* = 0.15) and for hypertension in 29% and 51% respectively (*p* = 0.02). No significant difference in QOL between groups and no difference in treatment‐related mortality.
Peterli R et al.^50^	Switzerland	Age 18–65, BMI >40 or >35 with significant comorbidity 25% have diabetes	45.5	72%	43.9 (43.6 v 44.2)	5 years	Diabetes remission, dyslipidaemia remission Gastric reflux remission.	Excess BMI loss was not significantly different at 5 years: for sleeve gastrectomy, 61.1%, vs. RYGB, 68.3%	Diabetes remission in 61.5% with sleeve gastrectomy and 67.9% with RYGB. (*p* = 0.22) Resolution of dyslipidaemia in 42.6% after sleeve gastrectomy and 62.3% (RYGB) –significant. Remission of reflux symptoms was in 25% in the sleeve gastrectomy group and 60.4% in RYGB; (*p* = 0.002).
Biter LU et al.^51^	Netherland	Age 18–65, BMI >40 or >35 with significant comorbidity 21.7% have diabetes	43	81.8%	43.5 (43.7 v 43.3)	5 years	Diabetes remission, dyslipidaemia remission, improvement in hypertension, improvement in sleep apnoea.	Excess BMI loss at 5 years was 58.8% after sleeve gastrectomy and 67.1% after RYGBypass (*p* < 0.001). Total weight loss at 5 years after sleeve gastrectomy and 26.0% after RYGB	Resolution of hypertension in 66.8% in the sleeve group vs. 73.8% in RYGB in the bypass group (*p* = 0.20). Resolution of Type 2 Diabetes in 55.6% with sleeve gastrectomy vs. 56.5% in RYGB. (NS) Improvement of dyslipidaemia in 62.0% after sleeve gastrectomy vs. 83.1% after RYGB, (*p* = 0.006). Sleep apnoea improved in 82% in the sleeve group, compared with 82.3% in the RYGB, (*p* = 0.93).
Hedberg S et al.^52^	Sweden, Norway	Adults (aged ≥18 years), BMI 35–50	42.9	73.9%	40.8 (40.8 v 40.9)	5 years	30 days peri‐operative outcomes, 90 day mortality	–	No 30 and 90 day mortality. Any adverse event occurred in 4.6% in the SG group and 6.3% in the RYGB group (*p* = 0.11).

Meta‐analyses performed to address the inconsistent results of empirical studies also produced varied conclusions. While two meta‐analyses report no significant difference in weight loss outcomes between the two procedures,^53^, ^54^ one outlines greater short‐term weight loss with RYGB,^55^ and another describes superior long‐term weight outcomes associated with RYGB.^56^ The recently completed ByBandSleeve trial is an important study in this field. The study randomized 1346 patients to receive adjustable gastric band, sleeve gastrectomy or RYGB.^57^ At the time of writing this review, the study has not been published, but preliminary data presented showed that RYGB produced the greatest mean weight loss and was associated with superior improvement in quality of life compared with the other procedures. Findings from this important study will provide guidance on the most appropriate MBS procedures to be performed in the absence of any contraindications between different MBS procedures.

Fortunately, with regard to remission of type 2 diabetes, the situation is much clearer as several studies have demonstrated significantly higher remission rates associated with RYGB in comparison with sleeve.^58^, ^59^, ^60^, ^61^ This finding is supported by meta‐analyses,^62^ although it has been suggested that this difference may not persist into the long term.^63^

OAGB is a relatively new bariatric procedure and, as such, data comparing outcomes between this and other types of surgery are limited. The results of two meta‐analyses suggest that OAGB results in superior weight loss to RYGB at 1 year, although this difference does not appear to persist past this point.^64^, ^65^ This superiority of OAGB was also reported in a meta‐analysis of comparisons between this and sleeve gastrectomy,^66^ although opposing results have been identified in other analyses.^42^ In addition, higher long‐term diabetes remission rates have been observed with OAGB versus sleeve gastrectomy and RYGB.^67^ However, the absence of high‐quality RCTs with long‐term follow‐up limits the ability for clear recommendations regarding OAGB to be made.^68^, ^69^ Similarly, the biliopancreatic diversion has demonstrated superior weight loss and diabetes remission in the limited number of comparative studies that have been performed, but further investigation is required, particularly due to the potential of a higher associated complication rate.^38^, ^70^

### 3.5 Endoscopic bariatric procedures

The last decade has seen the emergence of new endoscopic bariatric procedures (EBPs). The purported advantages are that these are less invasive and reversible. Three notable procedures are the intragastric balloons (IGB), endoscopic sleeve gastroplasty (ESG) and small bowel interventions such as the duodenal‐jejunal bypass liner.

IGB are space‐occupying devices designed to induce satiety, creating a sense of fullness, and delay gastric emptying. They are generally indicated for a lower BMI threshold of 30–40 kg/m^2^ and can be expected to achieve anywhere from 8 to 15% total body weight loss in the short term, depending on the type of balloon used.^71^, ^72^ IGB has also been increasingly utilized as a bridging intervention before definitive MBS procedures.^73^ Different types of intragastric balloons exist and vary in terms of material, single or multiple, duration of implantation, volume, adjustability and the methodology of insertion and removal. The three FDA‐approved balloons are the Obalon, Orbera and ReShape balloons. These three balloons are also approved in Europe, as well as the Elipse, End‐Ball, Heliosphere BAG, Lexbal, MedSil and Spatz3.^74^ Despite their non‐invasive nature, adverse events do occur. The most common complications include nausea, vomiting and abdominal pain in more than 20% of people, especially in the first week after placement when adaptation to the device is taking place. More serious complications such as obstruction, perforation or death can occur^75^; and hence patient selection and subsequent monitoring are crucial. Hyperinflation^76^ and acute pancreatitis^77^ have also been reported, but the latter has mainly been observed with liquid‐filled balloons.

**FIGURE 2:**
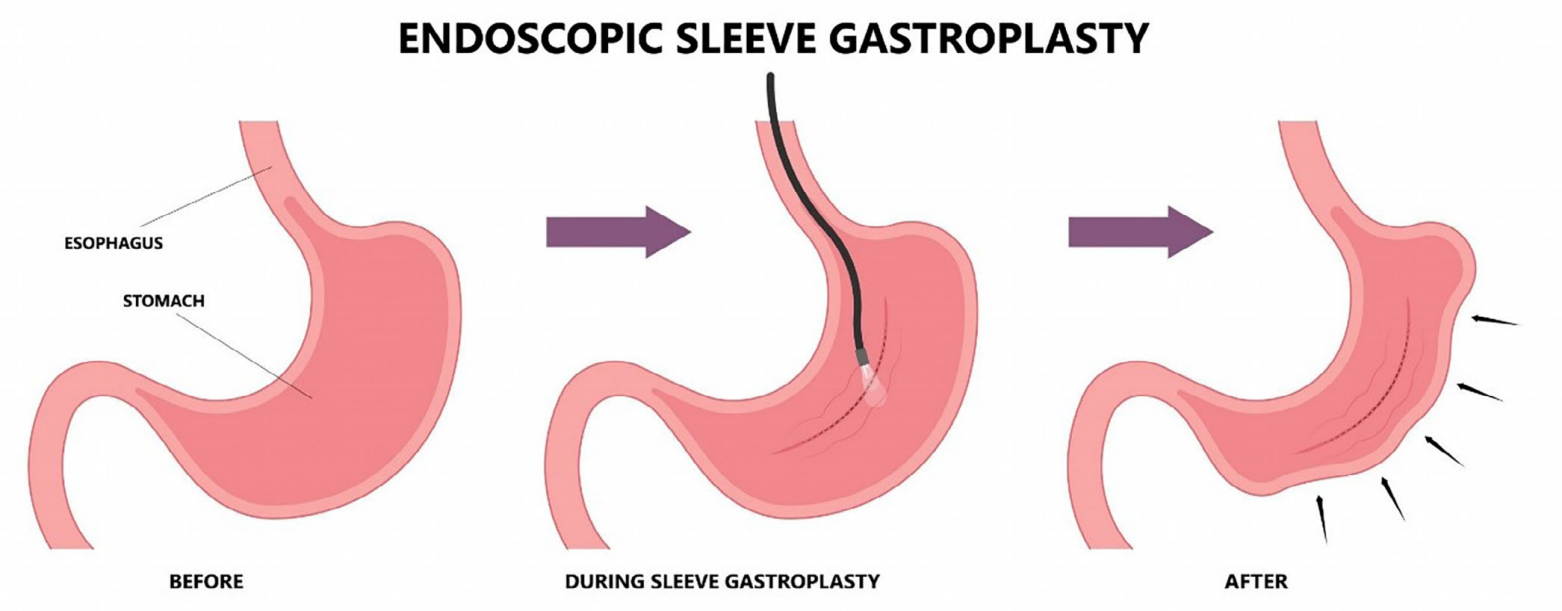
Endoscopic Sleeve Gastroplasty.

Endoscopic sleeve gastroplasty (ESG) is a minimally invasive endoscopic procedure performed under general anaesthesia that involves full‐thickness suturing of the stomach wall to create a longitudinal and anteroposterior reduction in gastric volume by approximately 70%^78^ (Figure 2). In the absence of formal guidelines, the American Society of Gastrointestinal Endoscopy (ASGE) has published preliminary recommendations for all endo‐bariatric therapies including ESG^79^ to be considered for patients with a BMI of 30 to 45 kg/m^2^ who have failed to lose or maintain weight with diet and lifestyle interventions alone. More recently in the United Kingdom, the National Institute of Clinical Excellence (NICE) has issued an interventional procedure consultation document and aims to produce a final interventional procedures document to be considered before guidance is issued to the UK National Health Service for clinical use.^80^ The efficacy of ESG has been examined by non‐randomized studies and a randomized study. The MERIT trial proved the superiority of ESG compared with lifestyle modifications alone for weight loss.^81^ The mean percentage of total body weight loss (TBWL) was higher in the ESG group compared with the control group (13.6% ± 8.0% vs. 0.8% ± 5.0%, *p* < 0.0001). A systematic review by Fehervari et al^82^ reported data from 35 studies involving a total of 7525 patients. Short‐term results were pooled from 23 studies (*n* = 5659) reporting an average TBWL at 1 year of 16.2% and 10 studies (*n* = 4040) reported medium‐term (at 3 years) TBWL of 15.4%.

Small bowel interventions include a duodenal‐jejunal bypass liner (Endobarrier) that functions as a malabsorptive device preventing contact of food substances with the intestinal mucosa. It anchors from the duodenal bulb, extends to the proximal jejunum and is removed at 12 months. A randomized trial including more than 300 people in the United States was stopped due to higher than anticipated rates of hepatic abscess, presumably related to portal hyperaemia induced by the device. In the worldwide EndoBarrier registry involving 1022 patients, the mean weight loss during EndoBarrier implantation was 13.3 kg (11.1% decrease in body weight from baseline), with associated improvements in glycaemic control, blood pressure and cholesterol.^83^ Importantly, limiting the implantation period to 9 months is likely to reduce the risk of hepatic abscess. In Europe, there is currently an application for restoration of the CE mark based on the new implantation period and evidence‐based gathered.^84^

Overall, these endoscopic procedures are new, induce modest weight loss compared with conventional bariatric surgery, and their outcomes are variable and have yet to be subjected to rigorous long‐term safety and efficacy assessment. Additional longitudinal studies and robust comparative trials are therefore required before these procedures can be widely utilized in routine clinical practice or incorporated into management guidelines.

### 3.6 Complications of MBS

Increased experience in MBS has resulted in a decline in perioperative risk of MBS—peri‐ and immediate post‐operative mortality is just under 0.1%,^85^, ^86^ while peri‐ and post‐operative morbidity varies widely depending on the type of surgery, patients' co‐morbidities and clinical demographics.^87^ While differences in mortality have not been observed between different MBS procedures, large national outcome studies of MBS procedures have reported increased likelihood for repeat intervention, endoscopy and hospital admission after RYGB compared with sleeve gastrectomy.^88^

The most common early post‐operative complications following MBS are intra‐operative leaks, stenosis, bleeding, venous thromboembolism, and respiratory distress and failure, the latter often due to undiagnosed obstructive sleep apnoea (OSA). In a study involving 135,000 patients who had RYGB or SG, the overall leak rate was 0.7%.^89^ Increased risk factors for developing post‐surgical leaks were oxygen dependency, hypoalbuminaemia, the presence of OSA, hypertension and type 2 diabetes.^89^ Early detection of leaks after MBS (e.g., intra‐operative endoscopy) is key to preventing major morbidity and mortality. Strategies to control leaks after MBS include the management of sepsis, drainage, the provision of enteral feeding and the prevention of distal obstruction such as stenosis in RYGB.

Long‐term post‐surgical complications include band migration, band erosion and access port infection for adjustable gastric band; stricture, reflux disease and fistula following SG; while marginal ulcers, anastomotic stenosis, internal hernia and candy cane roux‐syndrome are recognized complications following RYGB.^90^ The latter two complications often present as post‐prandial pain and occasional vomiting, with extreme cases leading to bowel ischaemia, which tends to occur late (>2 years) after the procedure.^90^ Intestinal obstruction may develop early or very late after surgery, while gallstone disease is also accentuated after MBS. Nutritional deficiencies and malabsorption can also occur, especially following RYGB.^90^, ^91^ Guidelines for monitoring and management of post‐operative vitamin and mineral deficiencies following MBS are described in Table 4.

**TABLE 4:** British Obesity, Metabolic Surgery Society (BOMSS) Guidelines for monitoring and management of post‐operative vitamin and mineral deficiencies following MBS.

	LAGB	RYGB	Sleeve Gastrectomy	LAGB	RYGB	Sleeve Gastrectomy
**FBC**	**X**	**X**	**X**			
**U&E**	**X**	**X**	**X**			
**LFT**	**X**	**X**	**X**			
**Ferritin**		**X**	**X**			
**Folate**		**X**	**X**			
**Calcium**		**X**	**X**			
**Vitamin D**		**X**	**X**			
**PTH**		**X**	**X**			
**Thiamine**		**S**	**S**			
**Vitamin B12**		**X**	**X**			
**Zinc**		**X**				
**Copper**		**X**				
**Vit A**		**S**				
**Vit E**		**S**				
**Vit K**		**S**				
**Selenium**		**S**				
**Multivitamin Supplement**				**X**	**X**	**X**
**Iron Supplement**					**X**	**X**
**Folic acid Supplement**					**X**	**X**
**Vit B12 supplement**					**X**	**X**
**Calcium & Vit D supplement**					**X**	**X**

*Note*: Annual screening blood test (first three columns) and Nutritional supplements (last three columns).

Abbreviations: LAGB, Laparoscopic gastric band; LFT, liver function test; PTH, parathyroid hormones S+ measure if concerning symptoms; RYG, Gastric bypass; U&E, urea & electrolyte.

The most common metabolic complication of MBS is dumping syndrome, with a prevalence of up to 40% of patients after RYGB or SG.^91^, ^92^ While most patients have mild symptoms, for some patients, symptoms of dumping syndrome can be debilitating. Early dumping typically occurs within the first hour after meals, manifesting as gastrointestinal symptoms (abdominal pain, bloating, borborygmi, diarrhoea) and vasomotor symptoms (flushing, palpitations, tachycardia, hypotension, fatigue or syncope) due to rapid introduction of hyperosmolar nutrients into the small bowel, causing fluid shift from the intravascular compartment to the intestinal lumen. Late dumping, meanwhile, occurs between 1 and 3 h after a meal, resulting in hypoglycaemia due to an incretin‐driven hyperinsulinaemic response after carbohydrate ingestion. Diagnosis is based on clinical symptoms and a positive modified glucose tolerance test based on the presence of an early (30 min) increase in haematocrit level >3% or pulse rate (>10 bpm) or the development of late (60–180 min) hypoglycaemia (glucose <2.8 mmol/L) after ingestion of a 75 g glucose load. Treatment is largely by dietary modification, delaying fluid intake until after 30 min after a meal, eliminating simple carbohydrates from the diet, consuming small and frequent meals consisting of high fibre, high protein in combination with complex carbohydrates, and the use of dietary supplements such as pectin with food. Pharmacological intervention is reserved for refractory cases and may include acarbose or somatostatin analogues such as octreotide, long‐acting somatostatin analogue, or more recently pasireotide,^92^ a multi‐receptor targeted somatostatin analogue.

Other additional metabolic complications following MBS that are often overlooked are osteoporosis and recurrent oxalate urolithiasis, both of which are most common after RYGB.^91^ In addition, weight regain can occur after MBS. Reasons are multifactorial, which include suboptimal dietary compliance, physiological reasons, concurrent drug use that may promote weight gain, or complications of MBS itself. Increased evidence over the last decade has also shown that MBS is associated with an increased risk of alcohol and substance use disorders.^93^, ^94^ Proposed mechanisms include increased peak blood alcohol concentrations after RYGB, changes in ghrelin responses, altered neural genetic expressions and changes in the reward responses in the brain.^95^ Studies have also shown that MBS is associated with increased suicide rates, risk of self‐harm and hospital admissions with depression.^96^, ^97^ Optimal psychological assessment is therefore an important part of patient preparation prior to MBS.

### 3.7 Pre‐operative management

Systematic identification, evaluation and optimal management of patients within a multi‐disciplinary setting prior to MBS is crucial, not only to reduce the peri‐ and post‐operative morbidity associated with MBS, but also to improve long‐term outcomes. Obesity is associated with a plethora of cardio‐respiratory‐related diseases such as coronary artery disease, atrial fibrillation, heart failure, hypertension, asthma and obstructive sleep apnoea—all of which require optimization and/or treatment prior to MBS.^98^ Achievement of optimal HbA1c (<69 mmol/mol) in people with type 2 diabetes has been shown to reduce risks of wound infection, prolonged hospital stay and acute kidney injury post‐operatively.^99^, ^100^ Rapid reduction of HbA1c following surgery may also potentially induce worsening of diabetic retinopathy.^101^ The decision to delay surgery and the appropriate threshold of HbA1c, however, should be individualized following discussion and agreement between surgeons, physician and anaesthetists. Assessment of drug history will highlight medications that may be associated with post‐operative complications (e.g., non‐steroid anti‐inflammatory drugs, corticosteroids, immunosupressions, anti‐coagulations or use of drugs which requires achieving optimal therapeutic index). Presence of significant GORD and hiatus hernia may impact on the type of MBS procedure to be performed (e.g., by avoiding sleeve gastrectomy).^102^ Identification of advanced cirrhosis prior to surgery is important to avoid the risks of developing peri‐ and post‐operative fulminant liver failure or variceal bleeding.^103^

MBS requires a life‐long commitment to specific eating behaviours and choices. Nutritional management therefore is important and involves assessment, education and treatment. Assessment includes information about previous weight loss attempts, social circumstances, cooking abilities, support networks, state of dentition and employment status to help highlight where support is needed. Education consists of guidance regarding regular meal patterns, portion size, macronutrient balance, reduced snacking and optimal fluid intake post‐surgery. Finally, nutritional treatment involves as a minimum, the provision of ‘liver shrinkage diet’ prior to MBS^104^ (usually achieve through total diet replacement) and improvement of nutritional balance post MBS to prevent future complications and deficiencies.^98^

As suggested previously, psychological assessment prior to MBS is important and aims to provide screening and identification of risk factors or potential post‐operative challenges that may contribute to poor post‐operative outcomes. Stevens et al^105^ published a clear traffic light illustration, outlining three levels of suitability for MBS, which has been supported by the Royal College of Surgeons, UK (Table 5). From these criteria, screening tools such as PHQ9 for depression, GAD7 for anxiety, eating problems and alcohol misuse (AUDIT‐C) can be utilized to identify suitability for surgery.

**TABLE 5:** Traffic light illustration of the three levels of suitability for bariatric surgery (Steven et al., 2012; ref. #^106^).

Red (not currently suitable for surgery)	Amber (possibly suitable, although deemed to be higher risk)	Green (suitable for surgery)
Unstable psychosis Active substance misuse and alcohol dependence Severe/moderate learning disability Dementia Severe personality disorder Self‐harm in past 12 months Active Bulimia Nervosa Current non‐adherence to treatment	Severe mental illness (mental state should be stable for 12 months with no hospital admission or self‐harm within that period History of alcohol or substance abuse History of an eating disorder Mild learning disability Poor motivation Unrealistic expectations Binge eating disorder Inadequate insight into eating behaviours Non –attendance Poor adherence to previous advice and treatment	Appropriate motivation Good understanding of procedures and outcomes Appropriate expectations Regular balanced diet Insight into eating and cases of weight gain Proven adherence to treatment

Patients undergoing MBS will pose problems with airway management and risks of brachial plexus injury. Pre‐operative anaesthetic assessment allows focus toward operative positioning while also assessing functional limitations and physiological reserve. Patients with obstructive sleep apnoea and/or obesity hypoventilation syndrome pose a specific risk to peri‐ and post‐operative complications.^106^ Identification of at‐risk patients and ensuring that patients are receiving optimal continuous positive airway pressure (CPAP) therapy leading to MBS are therefore crucial. Use of a risk calculator helps prognostication and facilitates decisions to appropriate high dependency care post‐surgery for high‐risk patients. The commonly used calculators are The American College of Surgeons (ACS) National Surgical Quality Improvement Programme (NSQIP) Risk Calculator (https://riskcalculator.facs.org/RiskCalculator), Nutritional Confidential Enquiry into Patient Outcomes and Death (NCEPOD) Surgical Outcome Risk Tool (SORT) v2 (https://www.sortsurgery.com) and the Physiological and Operative Severity Score for the Enumeration of Mortality and Morbidity (POSSUM score, http://www.riskprediction.org.uk/index-pp.php).

## 4 CONCLUSION

The escalating rise in the prevalence of obesity and type 2 diabetes has necessitated the need for aggressive multifactorial interventions to induce weight loss and reduce the long‐term complications of these diseases. Lifestyle interventions remain the mainstay treatment for obesity. This includes a variety of dietary and behavioural intervention approaches but with the main aim of inducing sustained energy restriction and increased levels of physical activity. MBS is now a highly effective intervention for long‐term weight management, which is increasing in prevalence globally. Sleeve gastrectomy and RYGB remain the most popular procedures, and more recent data appear to favour RYGB. The choice of procedure, however, will still rely upon several factors including surgeon preference and patient characteristics. Newer techniques such as OAGB and other endoscopic procedures have gained popularity and a growing evidence base, with the advantage of being less invasive and reversible. Preoperative assessment is crucial to reducing the risk of potential complications associated with MBS. Increased interest has also emerged in the role of pharmacotherapy to help weight loss for people living with obesity, although this is outside the remit of this review. Evidence based around pharmacotherapy for weight loss in tandem with lifestyle, dietary or even bariatric surgery interventions is covered in other chapters within this current journal supplement.

## CONFLICT OF INTEREST STATEMENT

The authors declare no conflicts of interest.

### PEER REVIEW

The peer review history for this article is available at https://www.webofscience.com/api/gateway/wos/peer-review/10.1111/dom.16296.

## Data Availability

The data that support the findings of this study are available from the corresponding author upon reasonable request.
